# Extra levothyroxine dose in Ramadan maintained normal thyroid hormone levels in patients with hypothyroidism: a randomized controlled trial

**DOI:** 10.3389/fendo.2025.1513904

**Published:** 2025-04-08

**Authors:** Nawal Al-Mutawa, Bashair M. Mussa, Suhair Akhlaq, Zeenat AbdulWahid, Ahmad Qawas

**Affiliations:** ^1^ Endocrinology Department, Al Qassimi Hospital, Emirates Health Services, Sharjah, United Arab Emirates; ^2^ Basic Medical Science Department, College of Medicine, University of Sharjah, Sharjah, United Arab Emirates; ^3^ Statistics and Research Center, Ministry of Health and Prevention, Dubai, United Arab Emirates; ^4^ Department of Endocrinology, Al Qassimi Hospital, Emirates Health Services, Sharjah, United Arab Emirates

**Keywords:** hypothyroidism, levothyroxine, fasting, Ramadan, TSH, FT3, FT4

## Abstract

**Introduction:**

The management of hypothyroidism during Ramadan represents a tangible challenge as levothyroxine (L-thyroxine), the first-line treatment for hypothyroidism, must be administered on an empty stomach at least 30 min before a meal in order to enhance its absorption.

**Aim:**

The present study aimed to compare the thyroid-stimulating hormone (TSH) levels among patients with hypothyroidism treated with an extra dose of l-thyroxine (25 mcg L-thyroxine, treatment group) *versus* a standard/regular dose (1.6 mcg/kg) of l-thyroxine (control group) during the month of Ramadan.

**Methods:**

This study is a randomized controlled clinical trial that included patients with hypothyroidism. Eligible participants (*n* = 103) were randomly allocated to the treatment group and the control group. Both groups attended five visits before, during, and after Ramadan. Several tests were conducted, including thyroid function, lipid profile, HbA1c, and vitamin D.

**Results:**

One of the most significant findings of the present study is that the extra dose of 25 mcg of L-thyroxine during Ramadan maintained the TSH levels of patients within the normal reference range, i.e., 0.55–4.78 mIU/L, at each visit during and after Ramadan without the need to wait 30 min before the meal. The mean TSH values were comparable between the treatment group and the control group during the five visits (visit 1, 3.00 ± 2.44 and 3.45 ± 3.02; visit 2, 3.62 ± 3.21 and 3.74 ± 2.74; visit 3, 4.19 ± 3.85 and 4.89 ± 2.92; visit 4, 3.54 ± 2.96 and 5.15 ± 4.26; and visit 5, 3.61 ± 3.05 and 3.32 ± 2.57, respectively).

**Conclusion:**

The present study demonstrated that the extra dose of L-thyroxine had a positive effect on keeping the TSH levels of patients in the normal reference range at each visit during and after Ramadan. However, in the control group, the mean TSH levels were higher than the normal range at visits 4 and 5.

## Introduction

Ramadan is the ninth month of the Islamic calendar, and millions of Muslims all over the world fast during this holy month. Ramadan fasting lasts 29 or 30 days based on the Islamic calendar (1). The month of Ramadan follows the Islamic calendar, which is 11–12 days shorter than the solar year; therefore, the dates for Ramadan change annually (2). Levothyroxine (l-thyroxine, LT4 hereinafter) is used for the treatment of hypothyroidism, which has a low cost and a few side effects (3). The standard dose of LT4 is 1.6 mcg/kg body weight, and it should be taken on an empty stomach at least 30 min before a meal. The absorption of LT4 decreases from 80% in fasting to 60% in the fed condition (4). Many patients fail to demonstrate a clinical and biochemical euthyroid status mainly due to non-compliance with LT4 therapy. During Ramadan, some patients take LT4 at Iftar (break of the fast, at sunset) with some water and then wait 30 min before eating their meal. This has been particularly challenging in recent years as fasting during Ramadan can be up to 15 h (5). There is great debate with regard to the best timing for the administration of LT4 during Ramadan. In certain special situations, it can be taken on an empty stomach while going to sleep or 3–4 h after the last meal (6).

A number of published studies have indicated that LT4 intake 1 h before Suhoor (the last meal before the fast, prior to sunrise) is suitable in the month of Ramadan. However, most patients had difficulty waking up that early and/or missed the dose and therefore took their medication together with the meals, which adversely affected its absorption (7–9). El-Kaissi et al. hypothesized that the post-Ramadan increase in the plasma thyroid-stimulating hormone (TSH) in the study would be differentially related to changes in the eating habits during Ramadan, leading to altered compliance with the administration instructions for LT4 (10). The plasma TSH began to increase as early as day 10 of Ramadan, peaking on day 26 and remaining significantly elevated 23 days post-Ramadan, with no significant changes in the free triiodothyronine (fT3) or the free thyroxine (fT4) levels. This retrospective analysis demonstrated that Ramadan is associated with a significant increase in plasma TSH, with approximately one-third of patients showing TSH values outside of the normal reference range post-Ramadan (10). The changes in plasma TSH were more prominent in older patients and in men, although the small male sample size was a limiting factor (10).

Adjustment in the LT4 dose is frequently required due to multiple factors, including inter-patient variations, LT4 absorption, change in the patient’s weight, and concomitant medication effects (11, 12). Taking LT4 on an empty stomach is necessary; however, after a prolonged duration of fasting, patient compliance with medication intake and waiting for at least 30 min between a meal and medication intake are extremely poor. Therefore, considering these findings, it is recommended to increase the dose of LT4 by 25 mcg/day to prevent variations in the thyroid function of fasting patients and to avoid the risk of influencing the quality of life. Although hypothyroidism is an extremely common condition in the United Arab Emirates (UAE), there are no previous studies conducted to evaluate the change in thyroid function by increasing the treatment dose of LT4 during Ramadan fasting in the UAE. Therefore, in the present study, it was hypothesized that fasting hypothyroid patients will require an increased dose of LT4 to maintain normal thyroid function during Ramadan. Accordingly, the present study was designed to compare the mean change in thyroid function among patients with hypothyroidism treated with an increased dose of LT4 (treatment group) *versus* those administered a standard/regular dose of LT4 (control group) during the month of Ramadan.

## Methods

This study is an open-label, two-arm, parallel-group randomized controlled trial conducted at the Endocrinology Clinic of the Family Promotion Centre (FPC) in Sharjah, which is one of the seven Emirates of the UAE. The study was approved by the Ministry of Health and Prevention, Research Ethics Committee (no. MOHAP/DXB-REC/MAO/no. 23/2021), and was conducted in accordance with the Declaration of Helsinki.

### Study design

This open-label, two-arm, parallel-group randomized controlled clinical trial included Emirati patients with hypothyroidism who regularly attended the Endocrinology Clinic of the FPC. Those who were interested in participating in the study were asked to sign an informed consent form (ICF) written in their native language. This form was approved by the Ethics Committee before recruitment.

### Study participants

**Figure 1:**
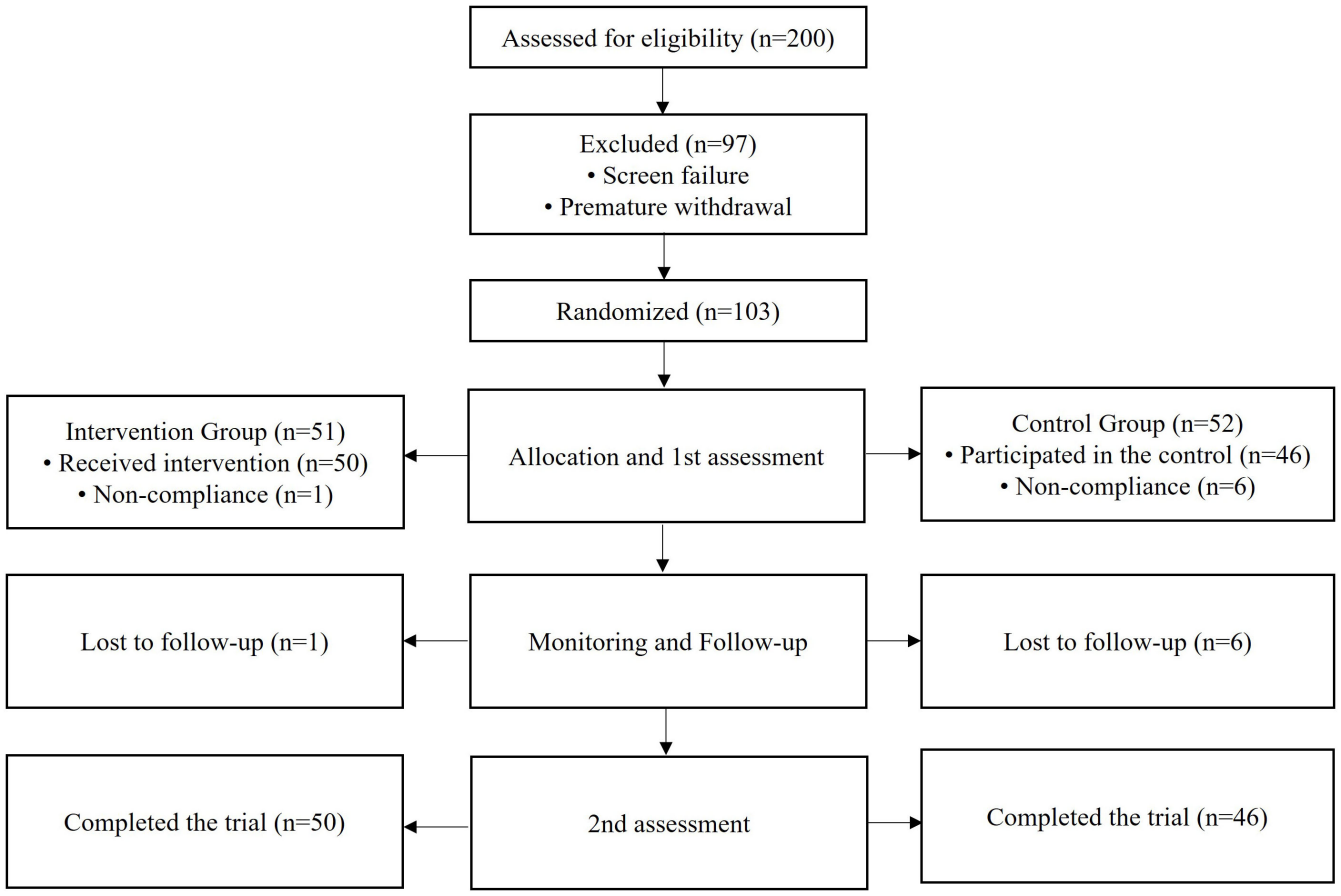
Schematic flowchart showing the selection process based on the inclusion and exclusion criteria.

Eligibility was assessed based on the inclusion and exclusion criteria. Screening was conducted on 200 Emirati potential participants, with approximately 97 participants who were excluded due to not meeting the criteria and to premature withdrawal (**Figure 1**). Eligible participants (*n* = 103) were randomly allocated into the treatment group (participants who received an increased dose of LT4, 25 mcg) and the control group (participants who received the standard dose of LT4). All participants were recruited from the Endocrinology Clinic of the FPC in Sharjah. Those who agreed to participate were asked to sign and date the ICF and were considered enrolled in the study upon submission of the signed ICF. Non-compliance and poor follow-up (*n* = 7) resulted in a minor reduction in the number of participants throughout the study. As shown in **Figure 1**, a total of 96 participants (treatment group: *n* = 50; control group: *n* = 46) completed the study and were included in the analysis.

The sample size included 103 cases. The 95% confidence interval (95%CI) was calculated, with 80% power of test and taking the expected mean ± SD of the mean change in TSH, i.e., 0.71 + 1.5 mIU/L (analyzed from a pilot study of 15 cases with an increased dose of LT4) *versus* 2.32 + 3.80 mIU/L (in the standard/regular group), among patients who presented with hypothyroidism using the sample size for the comparison of two means in the OpenEpi sample size calculator.

The inclusion criteria were as follows: Emirati (UAE citizens) male and female patients aged between 18 and 70 years with primary hypothyroidism; patients with stable TSH; and patients who regularly fast for at least 25–30 days during the month of Ramadan. The exclusion criteria were: patients with any end-organ damage; pregnant or breastfeeding women; and patients with thyroid cancer or any other diseases that could interfere with thyroxine absorption, including coeliac disease, inflammatory bowel disease, lactose intolerance, as well as *Helicobacter pylori* infection, and atrophic gastritis. In addition, patients with cardiovascular disorders, such as angina, coronary artery disease, and hypertension, were excluded. Moreover, patients who were not adhering to initial LT4 medications or those who were receiving proton pump inhibitory therapy, dietary fiber, bile acid sequestrates, ferrous sulfate, sucralfate, calcium carbonate, aluminum-containing antacids, phosphate binders, and raloxifene, were excluded. The exclusion criteria were comprehensive in order to avoid any confounding factors that might impact the analysis of the variables or the outcomes of the study.

### Study visits

**Figure 2:**
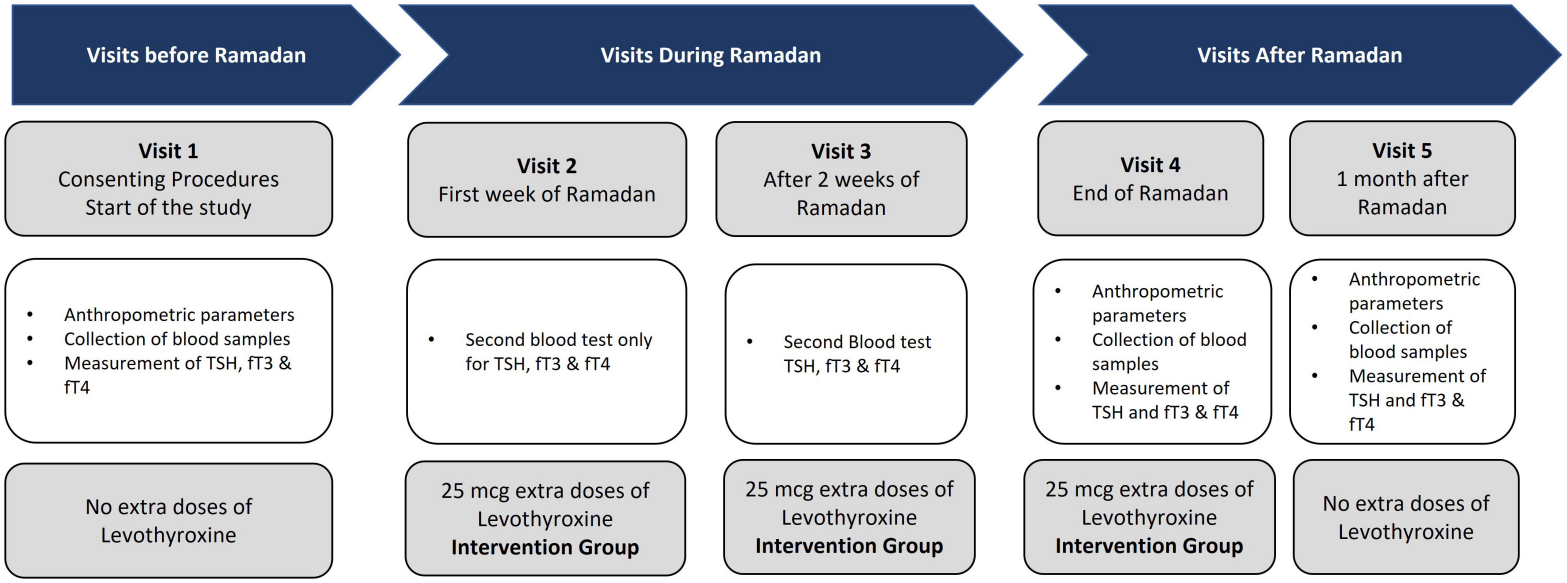
Schematic flowchart showing the site visits. Visit 1, start of the study; visit 2, first week after the start of Ramadan; visit 3, at 2 weeks after the start of Ramadan; visit 4, end of Ramadan; visit 5, at 1 month after Ramadan.

After randomization and allocation of the participants into the control group (those who received standard/regular dose of LT4) and the intervention group (those who received an increased dose of LT4, 25 mcg), all patients from the intervention and control groups had five site visits: the screening visit 14 days before Ramadan (visit 1), the second visit on the first week of Ramadan (visit 2), and the third visit conducted 14 days after the start of Ramadan month (visit 3). The fourth study visit was conducted 1 week after the end of Ramadan (visit 4), while the fifth visit was conducted 1 month after the end of the study (visit 5) (**Figure 2**).

At visit 1, the participants signed the ICF and started the study, during which all of the demographic, anthropometric, and clinical data were collected. In addition, blood samples were collected for the measurement of TSH, fT3, and fT4. No changes in the dose of LT4 were applied. At visits 2 and 3, blood tests were conducted only for TSH, fT3, and fT4, and patients in the intervention group started on an increased dose of LT4 (25 mcg). At visit 4, the anthropometric and clinical data were collected, as well as blood samples for the measurement of TSH, fT3, and fT4. The patients in the intervention group continued to receive the increased dose of LT4 (25 mcg). During the last visit (visit 5), the same protocol as that in visit 4 was followed, but the increased dose of LT4 was stopped.

### Statistical analysis

Data were entered and analyzed using SPSS statistical software, version 28. Continuous variables were summarized in terms of mean ± standard deviation and ranges. Categorical variables were presented as frequency and percentages. The chi-squared test was used to compare the categorical data in both groups, while the independent samples *t*-test, the paired samples *t*-test, and analysis of variance (ANOVA) were used to examine the differences between the thyroid function variables (i.e., TSH, fT3, and fT4) and the lipid profiles [i.e., total cholesterol (TC), high-density lipoprotein (HDL), low-density lipoprotein (LDL), and triglycerides] in both groups. A mixed-effects analysis was performed to identify the factors associated with the changes in the TSH, fT3, fT4, and cholesterol levels. A *p*-value ≤0.05 was considered statistically significant, and all hypothetical tests were used two-sided. Data were stratified with respect to the demographic and clinical characteristics using multivariate logistic regression. Odds ratios (ORs) >1 were considered significant.

## Results

The baseline characteristics of all the participants in the study are shown in **Table 1**. A total of 96 patients completed the study, who were classified into two groups: 50 patients in group I (extra dose) *vs*. 46 in group II (control). Most of the patients in both groups were in the age group 41–50 years [20 (40%) in group I *vs*. 20 (43.5%) in group II, *p* = 0.992]. There were no statistically significant differences in the demographic characteristics and the clinical variables, such as age, sex, marital status, education, body mass index (BMI), and duration of diagnosis, in both groups (**Table 1**). The majority of patients had autoimmune thyroiditis; six patients had hypothyroidism secondary to hypothyroidism secondary to radioactive iodine treatment for hyperthyroidism, while two patients had thyroidectomy secondary to goiter.

**Table 1:** Baseline demographic and clinical characteristics of the studied population.

Demographic Variables	Groups	Group I (Extra Dose of LT4) n (%)	Group II (Standard Dose of LT4) n (%)	P-value
***Age***	18-30 years	8 (16.0)	3 (6.5)	0.992
	31-40 years	9 (18.0)	11 (23.9)	
	41-50 years	20 (40.0)	20 (43.5)	
	51-60 years	13 (26.0)	6 (13.0)	
	61-70 years	0 (0)	6 (13.0)	
***Gender***	Male	2 (4.0)	5 (10.9)	0.196
	Female	48 (96.0)	41 (89.1)	
***Marital Status***	Single	8 (16.0)	7 (15.2)	0.621
	Married	41 (82.0)	39 (84.8)	
	Divorced / Widowed	1 (2.0)	0 (0)	
***Education***	Primary	2 (4.0)	3 (6.5)	0.816
	Secondary	12 (24.0)	12 (26.1)	
	College / Higher	36 (72.0)	31 (67.4)	
***BMI (Kg/m2)***	Normal weight (18.5kg/m2- 24.9 kg/m2)	7 (14.0)	3 (6.5)	0.476
	Overweight (25.0 kg/m2 to 29.9 kg/m2)	16 (32.0)	15 (32.6)	
	Obese (>30 Kg/m2)	27 (54.0)	28 (60.9)	
***Duration of Diagnosis***	</= 10 years	29 (58.0)	32 (69.6)	0.240
	> 10 years	21 (42.0)	14 (30.4)	

*LT4*, levothyroxine.

For the thyroid function test, the levels of TSH, T3, and T4 were reported at different visits and are shown in **Table 2**. Normal levels were observed in both the treatment and control groups at each visit. At baseline visit 1 (before Ramadan), the percentage of patients with normal levels was analyzed as treatment group I *versus* control group II, with results as follows: TSH, 80% *vs*. 82.6%; fT3, 85.1% *vs*. 81.8%; and fT4, 94.0% *vs*. 91.3%. The results after visit 4 (end of Ramadan) were as follows: TSH, 68.1% *vs*. 58.7%; fT3, 93.5% *vs*. 91.3%; and fT4, 97.9% *vs*. 95.7%. Due to the lack of statistical significance, an overly positive interpretation cannot be justified (**Table 2**).

**Table 2:** Comparison of the thyroid function (TSH levels) before, during, and after Ramadan.

Study group	Visit	TSH level				fT3 level				fT4 level			
		Normal, *n* (%)	Low, *n* (%)	High, *n* (%)	*p*-value	Normal, *n* (%)	Low, *n* (%)	High, *n* (%)	*p*-value	Normal, *n* (%)	Low, *n* (%)	High, *n* (%)	*p*-value
*Group I*	Baseline (visit 1)	40 (80)	3 (6.0)	7 (14.0)	0.642	40 (85.1)	7 (14.9)	0 (0)	0.673	47 (94.0)	3 (6.0)	0 (0)	0.545
*Group II*		38 (82.6)	1 (2.2)	7 (15.2)		36 (81.8)	8 (18.2)	0 (0)		43 (91.3)	2 (4.3)	1 (2.2)	
*Group I*	Visit 2	35 (70)	4 (8.0)	11 (22.0)	0.528	32 (65.3)	17 (34.7)	0 (0)	0.654	49 (98.0)	0 (0)	1 (2.0)	0.185
*Group II*		30 (65.2)	3 (6.5)	13 (28.3)		28 (60.9)	18 (39.1)	0 (0)		42 (91.3)	3 (6.5)	1 (2.2)	
*Group I*	Visit 3	28 (57.1)	5 (10.2)	16 (32.7)	0.234	39 (79.6)	9 (18.4)	1 (2.0)	0.35	47 (95.9)	1 (2.0)	1 (2.0)	0.353
*Group II*		26 (56.5)	1 (2.2)	19 (41.3)		41 (89.1)	5 (10.9)	0 (0)		43 (93.5)	3 (6.5)	0 (0)	
*Group I*	Visit 4	32 (68.1)	3 (6.4)	12 (25.5)	0.478	43 (93.5)	3 (6.5)	0 (0)	0.603	46 (97.9)	1 (2.1)	0 (0)	0.545
*Group II*		27 (58.7)	2 (4.3)	17 (37.0)		42 (91.3)	3 (6.5)	1 (2.2)		44 (95.7)	2 (4.3)	0 (0)	
*Group I*	Visit 5	36 (72.0)	3 (6.0)	11 (22.0)	0.726	45 (91.8)	4 (8.2)	0 (0)	0.259	49 (98.0)	1 (2.0)	0 (0)	0.496
*Group II*		35 (77.8)	3 (6.7)	7 (15.6)		40 (90.9)	2 (4.5)	2 (2.2)		43 (95.6)	2 (4.4)	0 (0)	

TSH, thyroid-stimulating hormone; T3, triiodothyronine; T4, thyroxine.

Furthermore, the percentage of patients with a normal lipid profile was analyzed as treatment group I *versus* control group II. The results of the analysis showed that the difference in the lipid profiles of patients during each visit before or after Ramadan between the extra LT4 group *versus* the control group (standard dose) was not statistically significant: TC, 68.0% *vs*. 63.0% (*p* = 0.690) at visit 1 and 72.3% *vs*. 56.5% (*p* = 0.111) at visit 4; HDL, 80.0% *vs*. 65.2% (*p* = 0.103) at visit 1 and 78.7% *vs*. 63.0% (*p* = 0.096) at visit 4; LDL, 74.0% *vs*. 69.6% (*p* = 0.629) at visit 1 and 73.9% *vs*. 63.0% (*p* = 0.262) at visit 4; and triglycerides, 88.0% *vs*. 87.0% (*p* = 0.877) at visit 1 and 76.6% *vs*. 82.6% (*p* = 0.472) at visit 4. The results are presented in **Table 3**.

**Table 3:** Comparison of the lipid profiles before, during, and after Ramadan.

Study group	Study visit	Cholesterol			HDL			LDL			Triglycerides		
		Normal, n (%)	High, n (%)	p-value	Normal, n (%)	High, n (%)	p-value	Normal, n (%)	High, n (%)	p-value	Normal, n (%)	High, n (%)	p-value
*Group I*	Baseline (visit 1)	34 (68.0)	16 (32.0)	0.609	40 (80.0)	10 (20.0)	0.103	37 (74.0)	13 (26.0)	0.629	44 (88.0)	6 (12.0)	0.877
*Group II*		29 (63.0)	17 (37.0)		30 (65.2)	16 (34.8)		32 (69.6)	14 (30.4)		40 (87.0)	6 (13.0)	
*Group I*	Visit 4	34 (72.3)	13 (27.7)	0.111	37 (78.7)	10 (21.3)	0.096	34 (73.9)	12 (26.1)	0.262	36 (76.6)	11 (23.4)	0.472
*Group II*		26 (56.5)	20 (43.5)		29 (63.0)	17 (37.0)		29 (63.0)	17 (37.0)		38 (82.6)	8 (17.4)	
*Group I*	Visit 5	38 (76.0)	12 (24.0)	0.589	35 (70.0)	15 (30.0)	0.145	40 (80.0)	10 (20.0)	0.607	43 (86.0)	7 (14.0)	0.195
*Group II*		32 (71.1)	13 (28.9)		25 (55.6)	20 (44.4)		37 (84.1)	7 (15.9)		34 (75.6)	11 (24.4)	

HDL, high-density lipoprotein; LDL, low-density lipoprotein.

At visit 5 (1 month after Ramadan), the patients were re-assessed, without an extra dose of LT4 in the intervention group, for thyroid function and the lipid profile. The results showed that the extra LT4 dose, once stopped, did not produce any significant effects on the TSH levels of the patients.

**Table 4** displays the mean values of the thyroid function test among patients during the different visits for the extra dose (group I) *versus* the standard LT4 dose (group II). It was observed that the extra dose (group I) of LT4 had a significantly positive effect on keeping the TSH levels of patients within the normal reference range of TSH (0.55–4.78 mIU/L) at each visit during Ramadan. However, in the control group (standard dose of LT4), during visits 3 and 4, the TSH levels were higher than the normal range.

**Table 4:** Mean TSH values before, during, and after Ramadan.

Study group	Visit	TSH results		fT3 level		fT4 level	
		Mean ± SD	p-value	Mean ± SD	p-value	Mean ± SD	p-value
*Group I*	Baseline (visit 1)	3.00 ± 2.44	0.463	4.20 ± 0.69	0.663	15.5 ± 3.26	0.711
*Group II*		3.45 ± 3.02		4.11 ± 0.82		15.6 ± 3.26	
*Group I*	Visit 2	3.62 ± 3.21	0.477	3.68 ± 0.57	0.808	15.5 ± 2.57	0.598
*Group II*		3.74 ± 2.74		3.60 ± 0.58		14.9 ± 2.79	
*Group I*	Visit 3	4.19 ± 3.85	0.091	4.05 ± 0.67	0.052	15.0 ± 2.84	0.056
*Group II*		4.89 ± 2.92		3.96 ± 0.42		14.0 ± 1.87	
*Group I*	Visit 4	3.54 ± 2.96	0.149	4.20 ± 0.43	0.154	15.6 ± 2.15	0.381
*Group II*		5.15 ± 4.26		4.36 ± 1.41		15.6 ± 2.37	
*Group I*	Visit 5	3.61 ± 3.05	0.357	4.25 ± 0.51	0.018	15.2 ± 2.25	0.956
*Group II*		3.32 ± 2.57		5.05 ± 3.48		15.7 ± 2.67	

TSH, thyroid-stimulating hormone; fT3, free triiodothyronine; fT4, free thyroxine.

The lipid profiles of the patients were assessed before Ramadan and before the initiation of the extra dose (visit 1), at the end of Ramadan with the extra LT4 dose in the intervention group (visit 4), and 1 month later (visit 5). The results showed that the total cholesterol, triglyceride, Hemoglobin A1C (HbA1C), and vitamin D3 levels were slightly increased at the end of Ramadan in both groups, but no statistically significant difference was observed at each visit (**Table 5**).

**Table 5:** Mean values of the lipid profile, HbA1C, and vitamin D before, during, and after Ramadan.

Study group	Visit	Cholesterol		HDL		LDL		Triglycerides		HbA1C		Vitamin D3	
		Mean ± SD	p-value	Mean ± SD	p-value	Mean ± SD	p-value	Mean ± SD	p-value	Mean ± SD	p-value	Mean ± SD	p-value
*Group I*	Baseline (visit 1)	5.00 ± 0.82	0.238	1.56 ± 0.33	0.340	2.95 ± 0.74	0.337	1.10 ± 0.45	0.183	5.75 ± 1.21	0.794	61.6 ± 26.3	0.424
*Group II*		4.96 ± 0.96		1.45 ± 0.35		3.00 ± 0.84		1.21 ± 0.60		5.87 ± 0.87		55.9 ± 18.5	
*Group I*	Visit 4	5.13 ± 0.95	0.060	1.49 ± 0.32	0.593	3.10 ± 0.80	0.054	1.29 ± 0.65	0.674	5.63 ± 0.93	0.438	56.0 ± 21.3	0.751
*Group II*		5.13 ± 1.13		1.41 ± 0.30		3.20 ± 1.03		1.22 ± 0.57		5.75 ± 0.92		57.8 ± 18.2	
*Group I*	Visit 5	4.81 ± 0.88	0.588	1.53 ± 0.36	0.938	2.87 ± 0.76	0.646	1.21 ± 0.57	0.301	5.80 ± 0.83	0.184	63.2 ± 25.4	0.953
*Group II*		4.72 ± 0.93		1.43 ± 0.35		2.91 ± 0.82		1.27 ± 0.60		5.95 ± 0.90		64.0 ± 22.5	

HDL, high-density lipoprotein; LDL, low-density lipoprotein; HbA1C, glycated hemoglobin.

As shown in **Table 6**, the paired samples *t*-test was applied to measure the mean difference in the serum TSH levels and the lipid profiles between pre- (visit 1) and post-Ramadan (visit 4). The TSH levels at visit 1 was 2.88 ± 2.45, which increased slightly to 3.54 ± 2.95 at visit 4, but did not reach statistical significance (*p* = 0.207). On the other hand, the fT3 (4.26 ± 0.62 *vs*. 4.20 ± 0.44, *p* = 0.499) and fT4 (15.63 ± 3.29 *vs*. 15.59 ± 2.14, *p* = 0.931) levels were found to be slightly decreased at the end of Ramadan with the extra LT4 dose, but with no statistical difference between the two groups. The serum cholesterol levels were slightly increased at visit 4 compared with those at baseline (4.99 ± 0.82 *vs*. 5.13 ± 0.95, *p* = 0.169), while HDL showed a significant decrease (1.56 ± 0.33 *vs*. 1.49 ± 0.32, *p* = 0.022). LDL showed a mild increase (2.95 ± 0.76 *vs*. 3.10 ± 0.80, *p* = 0.067), while triglycerides significantly increased at visit 4 (1.10 ± 0.46 *vs*. 1.25 ± 0.59, *p* = 0.021). HbA1c showed a mild decrease at the end of Ramadan in the intervention group (5.75 ± 1.26 *vs*. 5.63 ± 0.95, *p* = 0.240), and vitamin D3 significantly decreased at the end of Ramadan (62.12 ± 26.48 *vs*. 56.03 ± 21.27, *p* = 0.035).

**Table 6:** Paired group analysis for the mean change in the thyroid function and lipid profile before (visit 1) and at the end of Ramadan (visit 4).

	Mean change in the treatment group (increased dose of LT4)				Mean change in the control group			
	Mean ± SD	95% CI of the difference		p-value	Mean ± SD	95% CI of the difference		p-value
		Lower	Upper			Lower	Upper	
*TSH level at visit*1 − *TSH level at visit* 4	(−0.663) ± 3.55	−1.705	0.379	0.207	(−1.171) ± 5.012	−3.197	−0.220	0.025
*fT3 level at visit* 1 − *fT3 level at visit* 4	0.061 ± 0.588	−0.120	0.242	0.499	(−0.269) ± 0.156	−0.742	0.203	0.257
*fT4 level at visit* 1 − *fT4 level at* visit 4	0.041 ± 3.25	−0.911	0.994	0.931	0.046 ± 2.794	−0.784	0.875	0.912
*Cholesterol level at* visit 1 − *cholesterol level at* visit 4	(−0.139) ± 0.685	−0.3410	0.614	0.169	(−0.169) ± 0.657	−0.365	0.025	0.086
*HDL level at* visit 1 − *HDL level at* visit 4	0.064 ± 0.186	0.0096	0.1189	0.022	0.036 ± 0.165	−0.013	0.085	0.148
*LDL level at* visit 1 − *LDL level at* visit 4	(−0.150) ± 0.549	−0.312	0.110	0.067	(−0.197) ± 0.599	−0.354	−0.039	0.015
*Triglyceride level at* visit 1 − *Triglyceride level at* visit 4	(−0.153) ± 0.435	−0.282	−0.236	0.021	(−0.013) ± 0.458	−0.149	0.122	0.843
*HbA1C level at visit* 1 − *HbA1C level at* visit 4	0.114 ± 0.627	−0.0789	0.306	0.240	0.108 ± 0.361	0.000	0.217	0.051
*Vitamin D3 level at* visit 1 − *Vitamin D3 level at* visit 4	6.085 ± 18.96	0.454	11.715	0.035	(−1.822) ± 13.74	−5.950	2.306	0.379

CI, confidence interval; HDL, high-density lipoprotein; LDL, low-density lipoprotein; HbA1C, glycated hemoglobin; fT3, free triiodothyronine; fT4, free thyroxine.

In contrast, in the control group, there was a significant increase in the mean change in the serum TSH levels at the end of Ramadan compared with the baseline (3.45 ± 3.01 *vs*. 5.16 ± 4.26, *p* = 0.025). No significant difference in the mean change was observed for fT3, fT4, and TC, HDL, triglycerides, and vitamin D3. However, LDL was significantly increased and HbA1c was significantly decreased among patients at visit 4 (*p* < 0.05).

As shown in the results in **Table 7**, no statistically significant difference was found in the TSH, fT3, fT4, HDL, and triglyceride levels between visit 4 and visit 5 (1 month after Ramadan), with *p* > 0.05 in the intervention group. On the other hand, the cholesterol, LDL, and HbA1c levels were significantly increased 1 month after Ramadan (*p* < 0.05). Vitamin D was significantly improved after Ramadan (*p* < 0.001).

**Table 7:** Paired group analysis for the mean change in thyroid function and lipid profile at visit 4 (end of Ramadan) and at visit 5 (after Ramadan).

	Mean change in the treatment group (increased dose of LT4)				Mean change in the control group			
	Mean ± SD	95% CI of the difference		p-value	Mean ± SD	95% CI of the difference		p-value
		Lower	Upper			Lower	Upper	
*TSH level at visit* 4 − *TSH level at visit* 5	(−0.111) ± 3.59	−1.166	0.944	0.833	1.890 ± 3.45	0.852	2.929	0.001
*fT3 level at* visit 4 − *fT3 level at* visit 5	(−0.038) ± 0.48	−0.183	0.107	0.596	(−0.690) ± 3.70	−1.814	0.434	0.222
*fT4 level at* visit 4 − *fT4 level at* visit 5	0.048 ± 2.16	−0.187	1.084	0.162	(−0.177) ± 2.55	−0.945	0.590	0.644
*Cholesterol level at* visit 4 − *Cholesterol level at* visit 5	0.333 ± 0.75	0.112	0.555	0.004	0.443 ± 0.73	0.223	0.663	0.000
*HDL level at* visit 4 − *HDL level at* visit 5	(−0.043) ± 0.29	−0.129	0.042	0.307	(−0.009) ± 0.18	−0.063	0.046	0.739
*LDL level at* visit 4 − *LDL level at* visit 5	0.242 ± 0.52	0.089	0.397	0.003	0.358 ± 0.60	0.175	0.541	0.000
*Triglyceride level at* visit 4 − *Triglyceride level at* visit 5	0.113 ± 0.45	−0.019	0.247	0.092	(−0.032) ± 0.47	−0.174	0.110	0.651
*HbA1C level at visit* 4 − *HbA1C level at* visit 5	(−0.180) ± 0.47	−0.321	−0.039	0.013	(−0.216) ± 0.30	−0.307	−0.125	0.000
*Vitamin D3 level at* visit 4 − *Vitamin D3 level at* visit 5	(−7.369) ± 13.16	−11.28	−3.460	<0.001	(−7.781) ± 12.89	−11.850	−3.713	0.000

CI, confidence interval; LT4, levothyroxine; HDL, high-density lipoprotein; LDL, low-density lipoprotein; HbA1C, glycated hemoglobin; fT3, free triiodothyronine; fT4, free thyroxine.

However, in the control group, the TSH level, which was increased at visit 4, returned to normal at visit 5 (1 month after Ramadan), with a significant difference (1.890 ± 3.45, *p* < 0.001). The fT3 and fT4 levels were perceived to be slightly increased at visit 5, with non-statistically significant differences. The cholesterol level was improved significantly after Ramadan (0.443 ± 0.73, *p* < 0.001). The levels of HDL and triglycerides did not significantly differ between visit 4 and visit 5, but LDL was significantly decreased at visit 5 (*p* < 0.001). HbA1c was significantly increased [(−0.216) ± 0.30, *p* < 0.001]. The vitamin D3 level was significantly improved, with a *p*-value <0.001 at visit 5 in comparison to visit 4.

## Discussion

This study is a randomized controlled trial that examined the impact of increasing the LT4 dose (25 mcg) during Ramadan for patients with hypothyroidism. In patients using LT4, the effects of Ramadan fasting on the TSH were evaluated. Interestingly, there is no study evaluating the effect of the addition of an extra dose, i.e., 25 mcg, of LT4 on thyroid function during Ramadan fasting. This highlights the importance of the present study, which investigated for the first time the effects of the addition of an extra dose of LT4 for patients who fast during Ramadan.

One of the most significant findings of the present study is that the extra dose of 25 mcg LT4 during Ramadan had a positive effect on maintaining the TSH levels of patients within the normal reference range (0.55–4.78 mIU/L) at each visit during and after Ramadan. However, in the control group, i.e., patients who received the standard dose, the mean TSH levels at visits 3 and 4 were higher than the normal range, 4.89 + 2.92 and 5.15 + 4.26, respectively. It is noteworthy that further analysis of the TSH levels before and after Ramadan showed a statistically significant increase in the control group. The fluctuations in the TSH levels in the control group could be explained by the changes in gastric motility due to prolonged fasting and the relieving of hunger with heavy meals, which may have caused fluctuations in the circadian rhythm. In addition, the deiodinase activity, and consequently the LT4 metabolism, may have also been influenced (6, 13–17). Patients who fast for more than 15 h usually prefer to start eating with their family immediately, after sunset, in order to avoid prolonged hunger and thirst. This increases the risk of abnormal results, i.e., with high TSH, as the absorption of LT4 declines from 80% in the fasting condition to 60% in the fed condition. Therefore, the American Thyroid Association (ATA) recommends a 60-min interval between the intake of LT4 and eating or 3 h after the previous meal (1).

The study of Azizi et al. showed that, during Ramadan, proper administration of LT4 is achieved if it is taken either 1 h before Iftar or 1 h before Suhoor. However, most patients found it difficult to wait before Iftar or wake up earlier than the Suhoor time (18). Therefore, most of the patients missed the dose or took it with the meal in the fed condition. This problem can be easily managed if the extra dose of LT4 is taken at Iftar and the patients can have their meals with their family and not wait for longer. The findings of the present study supported this recommendation, indicating that patients can take an extra dose of LT4 and maintain the TSH levels within the normal range during Ramadan.

Previous studies investigated the impact of Ramadan fasting in well-controlled hypothyroid patients, with a sample size ranging from 112 patients in El-Kaissi et al. to 47 patients in Karoli et al. (10, 19). However, none of these studies examined the impact of increasing the LT4 dose during Ramadan. In addition, the limited data were obtained from a small number of studies published on hypothyroidism and Ramadan fasting that recommended increasing the LT4 dose by 25–50 mcg/day at the beginning of the month of Ramadan (20). This approach is believed to prevent fluctuations in the TSH levels of fasting patients. However, the effect of this intervention in the LT4 dose or the timing of its administration during Ramadan has not been evaluated using the present randomized controlled study design.

The absorption of LT4 is optimal during fasting (13, 14). However, there have been controversial findings with regard to the optimal administration time of this medication during Ramadan. Most physicians follow the recommendation of the ATA and the European Thyroid Association when prescribing LT4 during Ramadan and recommend one of the following timings: 30 min before Iftar, 3 h after Iftar, 30 min before Suhoor, or 3 h after Suhoor (6, 15, 16). Therefore, the present study can modify this by adding an extra dose of 25 mcg of LT4 at Iftar and patients can have their meals without waiting for much longer. El-Kaissi et al., in a randomized study, reported a significant decrease in the plasma TSH levels when measured within 6 weeks after Ramadan in 50 patients that took LT4 30 min before Iftar, with a compliance rate of 69.4% (16). In addition, the prospective study by Dabbous et al. showed a significant decrease in the serum T4 levels within 2 weeks after Ramadan (5). However, the present study demonstrated that a normal thyroid function was maintained during Ramadan and that the TSH levels were within the normal range after Ramadan in the intervention group, which had an extra dose of LT4.

Previous reports showed that T3 was reduced during malnutrition or starvation in normal subjects or in subjects with obesity for 4 weeks (21). Another study also revealed that the mean plasma T3 was reduced to approximately 50% due to starvation compared with the control (22). In another study, adult patients with protein–calorie malnutrition showed a reduction in plasma T3 to extremely low values (20% of normal; with re-feeding, T3 returned to or toward normal) (23). Moreover, the serum concentrations of T3, T4, and TSH were examined in seven men and seven women of normal weight during a 60-h fast, which demonstrated a decrease in circulating T3 (24). A 20% decrease in plasma T3 was also found by the end of the first day of fasting (24 h), while a 50% decrease was found at the end of 3 days of fasting (21). Karoli et al., in their study that included 49 patients, stated that LT4 should be taken after waiting for at least 2 h between a meal and taking the medication. However, it was determined in the same study that approximately 75% of the patients fasting during Ramadan did not wait long enough between the meal and the drug intake (19).

The outcomes of the published studies on hypothyroidism and Ramadan fasting recommend increasing the LT4 dose by 25–50 mcg/day at the beginning of the Ramadan month and continuing the increased dose for up to 15–20 days after Ramadan (20). This approach is believed to prevent fluctuations in the TSH values of fasting patients (20).

The present study also assessed other non-thyroid parameters, including the lipid profile, HbA1C, and vitamin D. Although most of the results were not statistically significant, a noticeable reduction was observed in HDL in the intervention group. The latter appears to be related to fasting rather than to the extra dose of TSH, as previous studies have reported a significant decrease in HDL due to changes in lifestyle habits, including lower levels of physical activity during Ramadan (25, 26). Moreover, increases in the LDL and triglyceride levels were observed during Ramadan in both groups. Although there were variations in the outcomes of the previous research with regard to the lipid profile during Ramadan, Sävendahl et al. elucidated that the increase in LDL during fasting was due to lipolysis and the decreased uptake of LDL by the liver (27). In agreement with previous findings, the present study demonstrated a reduction in HbA1C in response to fasting, and although several suggestions have been given to explain this reduction, it seems that sirtuin-6 proteins play a significant role in glycemic control as they enhance glucose homeostasis and decrease insulin resistance (28). Analysis of the HbA1C results showed that a reduction in the latter ranged between moderate to significant. This is in line with previous studies that demonstrated a 0.3% reduction in the mean Hb1Ac during Ramadan and an associated improvement in glycemic control (29). A significant reduction in vitamin D was observed during Ramadan; however, it improved 1 month after Ramadan. The association between low vitamin D levels and hypothyroidism has been previously observed and might explain the reduction in vitamin D (30). On the other hand, several studies revealed the positive effects of fasting on the levels of vitamin D in healthy participants (31).

### Limitations

This study has some limitations in terms of the small size of the studied population and the fact that it is a single-center study. In addition, evaluation of the medications for other comorbidities in the studied population was not performed. Moreover, the extra dose of LT4 was fixed for all patients and was not weight-based. As the present study is the first to address this research question, an extra high dose was avoided in order to prevent any complications that might have developed as a result. It is noteworthy that the small extra dose given to the patients was not for replacement but to avoid loss of the basic dose during the absorption of food. In future studies, we will be considering a weight-based dose adjustment of LT4.

## Conclusion

The present study demonstrated that the extra dose of LT4 had a positive effect on keeping the TSH levels of patients in the normal reference range at each visit during and after Ramadan. However, in the control group, the mean TSH levels were higher than the normal range during visits 4 and 5. Taking into consideration the challenges that hypothyroid patients face on a regular basis during Ramadan, the findings of the present study provided a more feasible solution, i.e., the addition of an extra dose of LT4 at Iftar time, without risking the normal thyroid function or the social commitment of having the Iftar with family on time.

## Data Availability

The original contributions presented in the study are included in the article/supplementary material. Further inquiries can be directed to the corresponding author.
